# Deciphering *Babesia*-Vector Interactions

**DOI:** 10.3389/fcimb.2017.00429

**Published:** 2017-09-29

**Authors:** Sandra Antunes, Catarina Rosa, Joana Couto, Joana Ferrolho, Ana Domingos

**Affiliations:** ^1^Global Health and Tropical Medicine, Instituto de Higiene e Medicina Tropical, Universidade Nova de Lisboa, Lisbon, Portugal; ^2^Instituto de Higiene e Medicina Tropical, Universidade Nova de Lisboa, Lisbon, Portugal

**Keywords:** tick-pathogen interaction, *Babesia* spp., vector, babesiosis, tick-borne diseases

## Abstract

Understanding host-pathogen-tick interactions remains a vitally important issue that might be better understood by basic research focused on each of the dyad interplays. Pathogens gain access to either the vector or host during tick feeding when ticks are confronted with strong hemostatic, inflammatory and immune responses. A prominent example of this is the *Babesia* spp.—tick—vertebrate host relationship. *Babesia* spp. are intraerythrocytic apicomplexan organisms spread worldwide, with a complex life cycle. The presence of transovarial transmission in almost all the *Babesia* species is the main difference between their life cycle and that of other piroplasmida. With more than 100 species described so far, *Babesia* are the second most commonly found blood parasite of mammals after trypanosomes. The prevalence of *Babesia* spp. infection is increasing worldwide and is currently classified as an emerging zoonosis. *Babesia microti* and *Babesia divergens* are the most frequent etiological agents associated with human babesiosis in North America and Europe, respectively. Although the *Babesia*-tick system has been extensively researched, the currently available prophylactic and control methods are not efficient, and chemotherapeutic treatment is limited. Studying the molecular changes induced by the presence of *Babesia* in the vector will not only elucidate the strategies used by the protozoa to overcome mechanical and immune barriers, but will also contribute toward the discovery of important tick molecules that have a role in vector capacity. This review provides an overview of the identified molecules involved in *Babesia*-tick interactions, with an emphasis on the fundamentally important ones for pathogen acquisition and transmission.

## Introduction

Parasites from the genus *Babesia* are responsible for causing an emerging zoonotic disease called babesiosis. Transmission occurs mainly through the bite of a *Babesia*-infected tick and, less commonly, by blood transfusion (Leiby, 2006; Ord and Lobo, 2015).

At least four Ixodidae genus are recognized as *Babesia* vectors: *Rhipicephalus, Ixodes, Haemaphysalis*, and *Hyalomma* (Sonenshine and Michael Roe, 2014). This disease has a considerable impact on the health and economy of the livestock industry, mainly in tropical and subtropical climates, with *Rhipicephalus microplus* and *Rhipicephalus annulatus* the main vectors of *Babesia bovis* and *Babesia bigemina*, the etiological agents of bovine babesiosis (Bock et al., 2004). In small ruminants, infections can be caused by several *Babesia* species, such as *B. ovis*, transmitted to sheep usually by the tick *R. bursa* (Shayan et al., 2007; Ranjbar-Bahadori et al., 2012; Ferrolho et al., 2016). Dogs are susceptible of infection by *B. canis vogeli* and *B. gibsoni*, primarily transmitted by *R. sanguineus* (Solano-Gallego et al., 2016; Chao et al., 2017). Human babesiosis, caused largely by *Babesia microti* and *Babesia divergens*, is not acknowledged as a tropical neglected disease, but there is a growing concern globally regarding this emerging zoonosis (Ord and Lobo, 2015).

Despite the fact that *Babesia* infections tend to impair tick development, an adaptive tolerance to *Babesia* has been described in *R. microplus* suggesting a balance between tick defense mechanisms and tick-pathogen mutual interaction(s) (Cen-Aguilar et al., 1998; Chauvin et al., 2009; Florin-Christensen and Schnittger, 2009; Lack et al., 2012; Gou et al., 2013; de la Fuente et al., 2016).

The development of improved tick and tick-borne disease control measures are essential to overcome the lack of data regarding which tick molecules are important and how they may be suitable as study targets. Based on this, herein we will discuss the functional roles of several molecules involved during the infection of tick tissues by *Babesia* spp.

## Tick midgut molecules with a role in *Babesia* acquisition

Once ingested *Babesia*-infected red-blood cells reach the tick midgut many parasites will be destroyed or degenerate, but a small number will evolve to gametocytes, essential for zygote fusion and penetration of the midgut peritrophic membrane (Sonenshine and Hynes, 2008; Chauvin et al., 2009; Maeda et al., 2017). Recently, it was proposed that during the *Babesia* spp. sexual phase, some specific proteins with known functional roles in recognition and adhesion are expressed, including glycosylphosphatidylinositol (GPI) anchored proteins that interact with specific targets in the epithelial cells (Bastos et al., 2013; Alzan et al., 2016).

In the *R. microplus* midgut, proteomic analysis has identified a mitochondrial voltage-dependent anion-selective channel (**BmVDAC**) polypeptide, also known as mitochondria porin that binds to *B. bigemina* sexual stage proteins (Mosqueda et al., 2004; Rodríguez-Hernández et al., 2012). VDAC was first described as located in the external mitochondrial membrane that regulates the flux of small molecules into the mitochondrial space membrane having a role in cell metabolism and apoptosis (Young et al., 2007). In mosquitoes, VDAC plays a role during *Plasmodium* sp. invasion of the midgut; likewise, the dissemination of *B. burgdorferi* through the tick midgut might be associated with the ability of VDAC to bind a tissue-type plasminogen activator (Coleman et al., 1997; Ghosh et al., 2011). Under *Babesia* invasion this protein was found over-represented in the *R. microplus* midgut (Rodríguez-Hernández et al., 2012).

The tick receptor of the outer surface protein A (**TROSPA**) was firstly identified in the *I. scapularis* midgut epithelium as a receptor for *B. burgdorferi*, suggesting it has the potential to control bacterial infections in ticks (Pal et al., 2004; Konnai et al., 2012; Urbanowicz et al., 2016). In *R. annulatus*, an orthologue of *trospa* gene was over-expressed during *B. bigemina* infection and gene knockdown significantly reduced *B. bigemina* infection levels by 70 and 83% in *R. microplus* and *R. annulatus*, respectively (Antunes et al., 2012). In addition, *B. bigemina*-infected cattle vaccinated with TROSPA revealed close to an 80% decrease in pathogen transmission to ticks (Merino et al., 2013). In *R. annulatus*, this receptor was found not only in the midgut, but also in the salivary glands (SGs) and ovaries (Antunes et al., 2014).

During protozoal invasion, the tick innate immune response leads to the rapid, synthesis of **defensins** and tick antimicrobial peptides (**AMPs**). These constitute an important humoral defense mechanism, which is also active against intracellular bacteria and fungi (Antunes et al., 2012; Hajdusek et al., 2013; Tonk et al., 2015). The midgut defensin-like protein, **longicin**, was first identified in the tick *Haemaphysalis longicornis* and has a role in *Theileria equi* proliferation (Tsuji et al., 2007). Merozoite *in vitro* cultures were inhibited in the presence of recombinant longicin while the inoculation of this protein led to a reduction of *B. microti* parasitaemia in infected mice. Also, *longicin* silencing led to an increase in *B. gibsoni* parasitaemia in several tick tissues, including midgut, ovaries and eggs. Accumulated data on the function of this protein indicate that longicin has a babesiacidal effect. **Microplusin** was the first fully characterized member of a family of cysteine-rich AMPs in *R. microplus* (Fogaça et al., 2004); in *R. annulatus*, was found over represented in response to *B. bigemina* infection (Antunes et al., 2012).

Other molecules present in the midgut that also protect the tick from pathogen invasion are the **MD-2-related lipid-recognition** (ML)-domain containing proteins related with lipid recognition (Rudenko et al., 2005), proteases and protease inhibitors (Sonenshine and Hynes, 2008; Kopacek et al., 2010; Antunes et al., 2012; Hajdusek et al., 2013). **Longipain**, a *H. longicornis* midgut cysteine protease, has shown similar effects to longicin. Recombinant longipain was also able to inhibit the proliferation of *T. equi* merozoites, and gene silencing resulted in an increase of protozoa in the midgut lumen, ovaries and hatched larvae (Tsuji et al., 2008). Also in *H. longicornis*, a **leucine-rich repeat domain-containing protein (LRR)** has been identified as over represented in all tick tissues, with the exception of the ovary, where it is constitutively expressed. *In vitro*, a specific recombinant LRR has demonstrated a growth inhibitory effect on *B. gibsoni* with similar or better results than traditional anti-babesial drugs (Maeda et al., 2015).

Tick **Kunitz-type protease inhibitors** may restrict pathogen infection, presumably via the inhibition of microbial proteinases (Sasaki and Tanaka, 2008; Antunes et al., 2012). This group of genes was upregulated in response to infection (Antunes et al., 2012; Heekin et al., 2013), but its influence in *Babesia* acquisition was only related to ovary infection (Rachinsky et al., 2007; Bastos et al., 2009).

**Bm86** is a glycoprotein, recognized for the first time in *R. microplus*, and present in midgut cells, that is likely to be involved in the endocytosis of the blood ingested by ticks (Gough and Kemp, 1993; Bastos et al., 2010; Rodríguez-Mallon, 2016). Regardless of the efficiency of Bm86 against tick infestation, some studies aimed to evaluate the role of Bm86 in *Babesia* infection (Bastos et al., 2010; Rodríguez-Mallon et al., 2013). RNA interference (RNAi) studies carried out in *R. microplus* females showed that *Bm86* silencing significantly reduced the number of ticks; by contrast, silencing did not affect the efficiency of transovarial transmission of *B. bovis* (Bastos et al., 2010). In a different study using Gavac®, a vaccine based on the Bm86 antigen, naïve nymphs that co-fed on immunized dogs presented lower levels of *B. canis*, (Rodríguez-Mallon et al., 2013). It is conceivable that the lysis of midgut cells inhibited the entry of zygotes and/or their posterior differentiation into motile ookinetes, compromising *B. canis* acquisition by the nymphs.

**Subolesin**, firstly identified in *I. scapularis* ticks as an orthologue of akirin in insects and vertebrates (Almazán et al., 2003; Galindo et al., 2009), is a highly conserved protein in eukaryotes, including many tick species (Moreno-Cid et al., 2013; Antunes et al., 2014), suggesting its potential as a candidate antigen for an anti-tick and tick-borne pathogen (TTBP) vaccine. Subolesin family proteins are transcriptional factors, regulating protein expression in cellular pathways involved in the response to pathogen infection (de la Fuente et al., 2013; Sultana et al., 2015). *Subolesin* silencing mediated by RNAi led to a lower *B. bigemina* infection in *R. microplus* (Merino et al., 2011) but, in contrast, in *R. annulatus*, silencing did not lead to a significant decrease in *B. bigemina* levels (Antunes et al., 2012). Vaccination using subolesin and a chimera containing subolesin protective epitopes (Q38) revealed an effect on *B. bigemina* transmission to feeding ticks (Merino et al., 2013). *Subolesin* expression and subolesin-mediated innate immunity varies according to the pathogen and tissue (Zivkovic et al., 2010), which explains the variation in the results. However, it seems that targeting subolesin by vaccination or its gene by RNAi would result in lower *Babesia* infection levels.

The tick midgut is one of the few major organs that defines vector competence since it is the first obstacle that several pathogens, including *Babesia*, have to cross. Still, our understanding of the interplay between an infective pathogen and the tick midgut continues to be poor and requires further studies to better define this important interaction.

## Tick haemolymph and ovary molecules acting in *Babesia* dissemination

After the successful invasion of the midgut epithelium, *Babesia* zygotes go through meiosis and differentiate into motile ookinetes that go across the haemocoel, with the help of haemolymph; in the haemocoel, the parasite undergoes asexual reproduction, resulting in several sporokinetes spread for all tick organs throughout all tick life stages (transstadial transmission) (Schnittger et al., 2012).

When a tick experiences microbial invasion, for example from a protozoa like *Babesia* spp., the hemocytes increase their circulating number to destroy and control the invader, phagocytizing small particles and microbes (Inoue et al., 2001; Villar et al., 2015). Besides phagocytosis, other processes including nodulation and encapsulation, and molecules like AMPs, lysozymes, proteases, protease inhibitors, and lectins, that exist in the haemolymph act directly on the pathogen (Esteves et al., 2008; Kotsyfakis et al., 2015). *B. bigemina* exhibits motility when reaching the haemolymph and adheres to *R. microplus* haemocyte membranes (de Rezende et al., 2015), however there is no information about how *Babesia* spp. invasion is controlled at the haemolymph level.

In female ticks, effective infection of ovaries and the eggs allow transovarial transmission of almost all *Babesia* species, a distinctive characteristic of this genus (Homer et al., 2000; Chauvin et al., 2009) that can be interpreted as an adaptation to efficiently persist in the ecosystem (Chauvin et al., 2009). The first ovarian proteomic profile of *R. microplus* infected with *B. bovis* identified a small number of differentially represented proteins. Among these proteins were calreticulin, glutamine synthetase and a family of Kunitz-type serine protease inhibitors; whereas between the less represented proteins were a tick lysozyme and a group of small proteins that may belong to a family of AMPs (Rachinsky et al., 2007). Ovarian genes involved in the stress response, detoxification and immune responses were found potentially regulated by *B. bovis* infection (Heekin et al., 2013); many of these genes translate into proteases and protease inhibitors that participate in the ovarian immune response. A putative *immunophilin* (*Imnp*) and a putative *Kunitz-type serine protease inhibitor* (*Spi*) genes were found to be up regulated when tick ovaries were infected (Rachinsky et al., 2007) and the *Imnp* knockdown revealed a significant increase of larval infection, suggesting that this molecule might control the protozoan invasion of tick ovaries, and subsequent larval progeny. **Immunophilin** proteins, also known as cyclophilins, are associated with multiple cellular processes, like protein folding, trafficking and defense mechanisms (Wang and Heitman, 2005), however their role(s) during *Babesia* infection is still unknown.

The *H. longicornis* **vitellogenin receptor** (VgR) has been associated with the transovarial transmission of *B. gibsoni*. *VgR* silencing results in the absence of *B. gibsoni* infection and development of abnormal eggs (Boldbaatar et al., 2008) confirming its influence on oogenesis acting on heme detoxification and egg maturation (Boldbaatar et al., 2010; Perner et al., 2016). These results may suggest that *Babesia* molecules have ligand-binding activity for tick VgR, consequently invading the developing oocyte (Boldbaatar et al., 2008).

Ovarian proteins can affect tick biology by decreasing oogenesis and embryogenesis, which reduce tick reproduction rates and TBP transmission by blocking transovarial transmission, making these molecules promising targets for vaccine development.

## Tick salivary gland molecules that intervene in *Babesia* transmission

When *Babesia* kinetes reach the SGs they undergo a final step of multiplication to produce sporozoites, the vertebrate host-infective stage. SGs can be considered as the last barrier that parasites must overcome to complete their life cycle in the vector, facing similar obstacles to those of the midgut (Chauvin et al., 2009).

Different SGs transcriptomes, commonly referred to as sialomes, from soft and hard ticks have been published (Francischetti et al., 2008, 2011; Anatriello et al., 2010; Karim et al., 2011; Ribeiro et al., 2011; Garcia et al., 2014; Yu et al., 2015; de Castro et al., 2016), showing genes encoding AMPs, such as defensins, microplusin/hebraein, Kunitz domain-containing proteins, lipocalins, proteases and other molecules related to tick defense mechanisms. Despite their importance for transmission, reports describing the influence of SG molecules on *Babesia* infection are absent.

The sialome of the soft tick *Ornithodoros parkeri* contains a putative **serum amyloid A** protein, whose orthologue was also found in the *I. scapularis* genome. In vertebrates, this protein is involved in the acute phase of an inflammatory response (Francischetti et al., 2008; Antunes et al., 2012). Vertebrate serum amyloid A protein was found increased in cattle with more resistance to tick infections, suggesting its involvement in the stress response induced by tick infestations (Ferreira et al., 2004). The expression of a putative *serum amyloid A* gene was increased in response to *B. bigemina* infection in *R. annulatus* and gene knockdown resulted in a reduction of 66 and 86% of the infection levels, in *R. microplus* and *R. annulatus*, respectively (Antunes et al., 2012).

**Calreticulin**, has been identified in tick ovaries, midgut and SGs (Antunes et al., 2012, 2015). The role of this molecule in ticks is still not clear but some studies support its presence in the SGs and saliva is presumably related to a mechanism to avoid vertebrate host defense responses (Jaworski et al., 1995; Ferreira et al., 2004; Antunes et al., 2015) and may lack the anti-thrombotic and complement-inhibiting characteristics that suppress host defense actions (Kim et al., 2015). The gene encoding this protein was found to be over expressed in *R. annulatus* infected with *B. bigemina. Calreticulin* knockdown had a significant effect on pathogen infection in *R. microplus*, but not in *R. annulatus* ticks, affecting the body weight in both tick species (Antunes et al., 2012). According to this and to other reports, it is thought that calreticulin acts during blood feeding (Ferreira et al., 2002; Antunes et al., 2012) and may alter calcium metabolism during *Babesia* infection. *Babesia* sp. may need calcium ions to invade tick cells as shown for *T. equi* (previously classified as *B. equi*). A pilot immunization trial in cattle using recombinant calreticulin failed to reduce tick infestation, probably due to the low immunogenicity of the protein (Ferreira et al., 2002). More recently, serum with anti-calreticulin antibodies also failed to promote a significant decrease in *B. bigemina* infection in *R. microplus* (Antunes et al., 2015). In this study, calreticulin immunolocalization assays have shown that this molecule can be found in the tick midgut, ovaries and SGs, suggesting that it might have a role in *Babesia* infection in all these tissues.

Other molecules, such as TROSPA, already discussed, have been also identified in tick SGs, where it may function as a receptor for *Babesia* parasites. Tick SG proteins are of extreme importance during *Babesia*-vector-host interactions and it seems likely that more molecules will emerge as key players in these vector-parasite networks in the near future.

**Figure 1:**
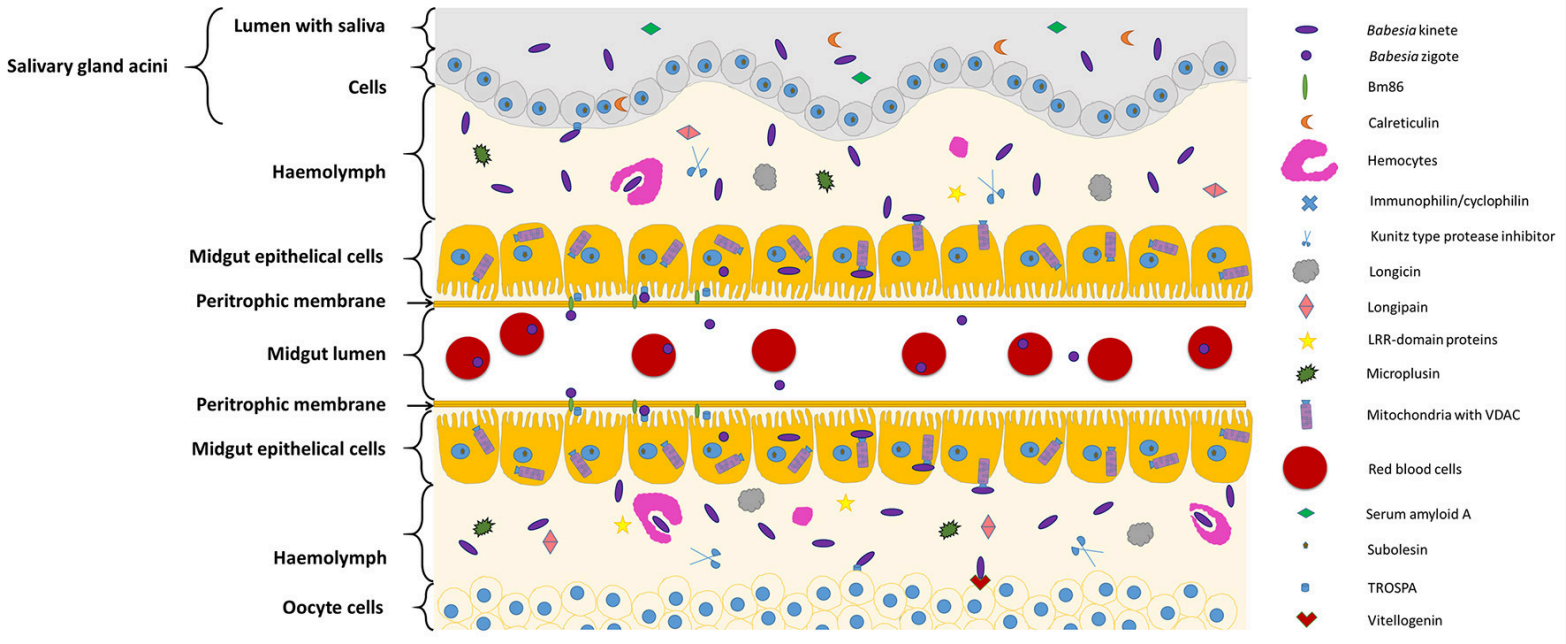
Diagram representing tick molecules implicated in *Babesia* spp. acquisition and transmission by the vector. When ticks feed on *Babesia*-infected animals, parasites within red blood cellsreach and penetrate the tick midgut peritrophic membrane to invade the epithelial cells (in the figure center). Once these cells are infected, transcriptional factors, such as subolesin, can regulate protein expression in several cellular pathways, facilitating *Babesia* infection. In the microvilli of the midgut cells, parasite zygotes will probably interact with a tick glycoprotein (Bm86) and a tick receptor of the outer surface protein A (TROSPA). Inside the epithelial cells, mitochondria porins (VDAC) can bind to *Babesia* kinete proteins promoting plasminogen activation in the cell surface, allowing their passage to the haemolymph. Once here, the haemocytes can phagocyte circulating parasites and the tick antimicrobial molecules such as, longicin, micropulsin, longipain, LRR-domain and Kunitz-type protease inhibitors are activated potentially reducing the infection in the vector. If the infectious parasites surpass these barriers of defense, they will be capable to spread across the tissues and invade ovaries (represented in the bottom of the figure) and SGs (represented in the top of the figure). In the ovary, the interaction of *Babesia* molecules with tick vitellogenin and TROSPA receptors may contribute for the occurrence of transovarial transmission; while in the SGs, *Babesia* interacts with TROSPA and calreticulin.

Figure 1, Table 1 summarizes the so far identified tick molecules networking with *Babesia* spp. showing their localization and suggested interaction.

**Table 1:** Tick molecules interfering with *Babesia* spp. infection.

**Protein name**	**Assigned function**	**Proteins localization**				**Described effect in *Babesia* spp. infection**	**References**
		**MD**	**HL**	**OV**	**SG**		
Mitochondrial voltage-dependent anion-selective channel (BmVDAC)	Cell metabolism and apoptosis regulation	X				Enhance acquisition	Rodríguez-Hernández et al., 2012, 2015
Tick receptor of the outer surface protein A (TROSPA)	Factor for tick gut colonization	X		X	X	Enhance acquisition	Antunes et al., 2012; Merino et al., 2013; Urbanowicz et al., 2016
Longicin	Defensin	X				Control acquisition and transovarial transmission	Tsuji et al., 2007
Microplusin	Antimicrobial peptide	X					Antunes et al., 2012
Longipain	Cysteine protease	X					Tsuji et al., 2008
Leucine-rich repeat domain-containing proteins	Component of the innate immune system	X	X	X	X	Control infection	Maeda et al., 2015
Kunitz-type protease inhibitors	Blood coagulation	X			X	Enhance transovarial transmission	Rachinsky et al., 2007; Bastos et al., 2009; Antunes et al., 2012
Bm86	Blood coagulation and cell growth	X		X		Enhance acquisition	Bastos et al., 2010
Subolesin	Transcriptional factor involved in the immune signaling pathways	X				Enhance acquisition	Merino et al., 2011; de la Fuente et al., 2013
Immunophilin	Protein folding, trafficking and defense		X			Control transovarial transmission	Rachinsky et al., 2007; Wang and Heitman, 2005
Vitellogenin receptor (VgR)	Vitellogenin uptake			X		Enhance transovarial transmission	Boldbaatar et al., 2008, 2010
Serum amyloid A	Response to inflammation	X				Enhance acquisition	Antunes et al., 2012
Calreticulin	Protein folding and signaling	X		X	X	Enhance acquisition.	Antunes et al., 2012

*MD, Midgut; HL, Haemolymph; OV, Ovaries; SG, Salivary Glands*.

## Conclusions

The major critical point for the development of vaccines is the identification of new targets. In this review, our objective was to gather relevant information about the tick molecules involved with *Babesia* parasite infections. During the last decade, several studies using “omics” and systems biology approaches have greatly improved our knowledge of the interactions taking place at the tick-pathogen interface. The *Babesia*-tick interactome is still neglected with scattered information, and only a few tick proteins have been shown to influence the acquisition, dissemination and transmission of the parasite. From this short list, subolesin, having a role in the tick innate immune response, stands out as a potential candidate antigen for a universal anti-vector vaccine. During *Babesia* infection, this molecule produced positive results, making it a candidate antigen for a transmission-blocking vaccine. Other proteins involved in *Babesia* acquisition, including the TROSPA receptor, are also promising candidates for a multi-antigenic vaccine. Some of these datasets were obtained through use of transcriptomic, proteomic, and systems biology approaches. These and future technologies will be fundamental to the improvement and development of new control strategies and more effective vaccines.

## Author contributions

SA, JF, CR, and AD conducted the literature research and wrote the paper. SA and CR prepared the table and JC prepared the figure. All authors critically read and revised the manuscript.

### Conflict of interest statement

The authors declare that the research was conducted in the absence of any commercial or financial relationships that could be construed as a potential conflict of interest.
